# A data set of bloodstain patterns for teaching and research in bloodstain pattern analysis: Impact beating spatters

**DOI:** 10.1016/j.dib.2018.02.070

**Published:** 2018-03-03

**Authors:** Daniel Attinger, Yu Liu, Tyler Bybee, Kris De Brabanter

**Affiliations:** a Mechanical Engineering, Iowa State University, 50010 Ames, IA, USA; b Department of Computer Science, Iowa State University, 50010 Ames, IA, USA; c Department of Statistics, Iowa State University, 50010 Ames, IA, USA

## Abstract

This is a data set of 61 blood spatter patterns scanned at high resolution, generated by controlled impact events corresponding to forensic beating situations. The spatter patterns were realized with two test rigs, to vary the geometry and speed of the impact of a solid object on a blood source – a pool of blood. The resulting atomized blood droplets travelled a set distance towards a poster board sheet, creating a blood spatter. Fresh swine blood was used; its hematocrit and temperature were measured. Main parameters of the study were the impact velocity and the distance between blood source and target sheet, and several other parameters were explored in a less systematic way. This new and original data set is suitable for training or research purposes in the forensic discipline of bloodstain pattern analysis.

**Specifications Table**

**Table:** 

Subject area	*Legal Medicine, Engineering*
More specific subject area	*Forensics – Bloodstain Pattern Analysis (BPA)*
Type of data	*Images of 61 blood impact beating spatters, with text file describing the experimental conditions*
How data was acquired	*Blood atomized with custom test rigs, as described in the manuscript. Spatters collected on poster board or butcher paper, and scanned with flatbed scanner A3 Epson Expression 11000XL*
Data format	*images scanned at 600 dot per inch (about 42.3 μm per pixel) and saved as.jpg with minimum compression (10% compression max)*
Experimental factors	*Spatter images with dimensions of max 1.36* *m × 1.1* *m, scanned in a piecewise manner and reassembled with image processing software*
Experimental features	*Blood pool of ~ 1* *mL volume on flat immobile surface impacted by either a cylindrical rod or a flat surface; impact velocities between 2* *m/s and 8* *m/s; horizontal distances between target and blood source from 30* *cm and about 2* *m; few data items involve multiple impacts on same target, and impact on soaked foam rather than pool*
Data source location	*Ames, IA. Physical Targets have been preserved*
Data accessibility	*Electronic data set is with this article, in two zip files of*Supplementary material

**Value of the data**

• The data set can be used by researchers to test crime scene reconstruction models [1], [2], [3]. Briefly, these models pursue two purposes. First, they classify patterns with respect to their generation mechanism (e.g. beating vs. shooting [4]). Second, they determine the region of origin of the blood spatter [5], [6]. Recently, the US National Academies emphasized [7] the need to develop more accurate bloodstain pattern analysis methods, with stronger fluid dynamics foundations. Accessibility to large amounts of bloodstain patterns produced under controlled conditions is thus important for the development of the needed science base [3], [7], [8], [9]. The data in this manuscript addresses the above issues by systematically documenting the experimental conditions.
• The data helps dissemination of blood spatters for teaching and instructional purposes. Indeed, production and transport of blood spatters is cumbersome. A large space, the size of a habitation room, is needed to create a realistic blood spatter; and care should be taken to have a reproducible and realistic generation mechanism, be it a beating or a gunshot. Blood sourcing and handling is not trivial either. Human blood for instance needs to be used under strict safety conditions because of the risk of blood-borne diseases and pathogens. Since travelling across borders is common for BPA instructors, both alternatives of travelling with blood spatters through customs or having the blood spatters prepared at the site and time of the workshop involve logistic efforts and safety risks. This database provides provide BPA instructors with a safe set of spatters ready to be printed for their classes.
• This data set is new and original, and has not been published elsewhere.
• The experimental design and methods described in this manuscript can be readily reproduced and used to create additional blood spatters.

## 1 Data

Blood spatters are a subset of bloodstain patterns, with stains generated by drops gone airborne [1], [2], [3] before hitting the surface of a solid object called the target. Impact spatters are spatters where blood is atomized by a force applied impulsively to the blood source [2]. Those features of atomization and airborne transport are rarely observable in a crime scene, and distinguish spatters from other bloodstain patterns, such as transfers where stains are produced by contact between the blood source and the target.

**Fig. 1:**
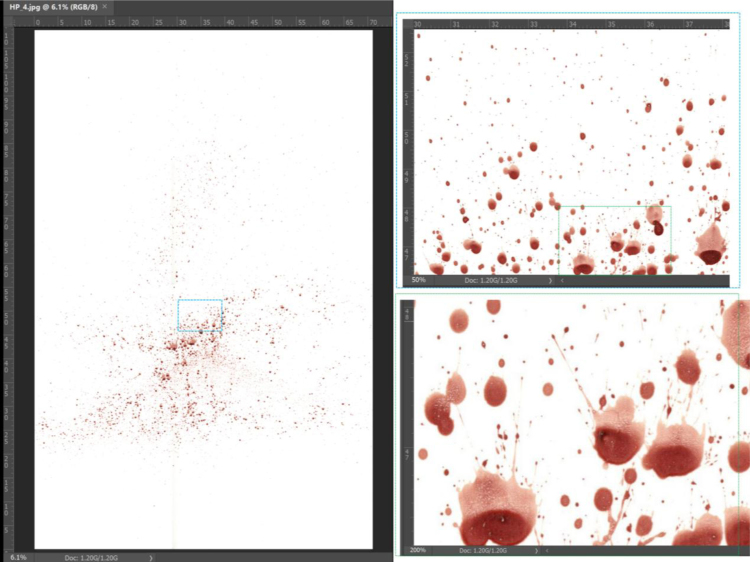
Example of blood spatter HP_4, with scale (in cm) indicated on the inset edges. The size of the target poster board is 70 × 110 cm, left. The high resolution of the stain edges is well visible. Image segmentation software such as the one used in [9] can count and measure more than 36,000 individual spots in the image, within a few minutes of processing time.

Here, two types of impact events are exerted in a controlled manner on a pool of blood to generate spatters representative of beating events. The scanned images of the spatters are provided as Supplementary material. Some of the spatters contain well above 1000 stains, as that in Fig. 1.

**Fig. 2:**
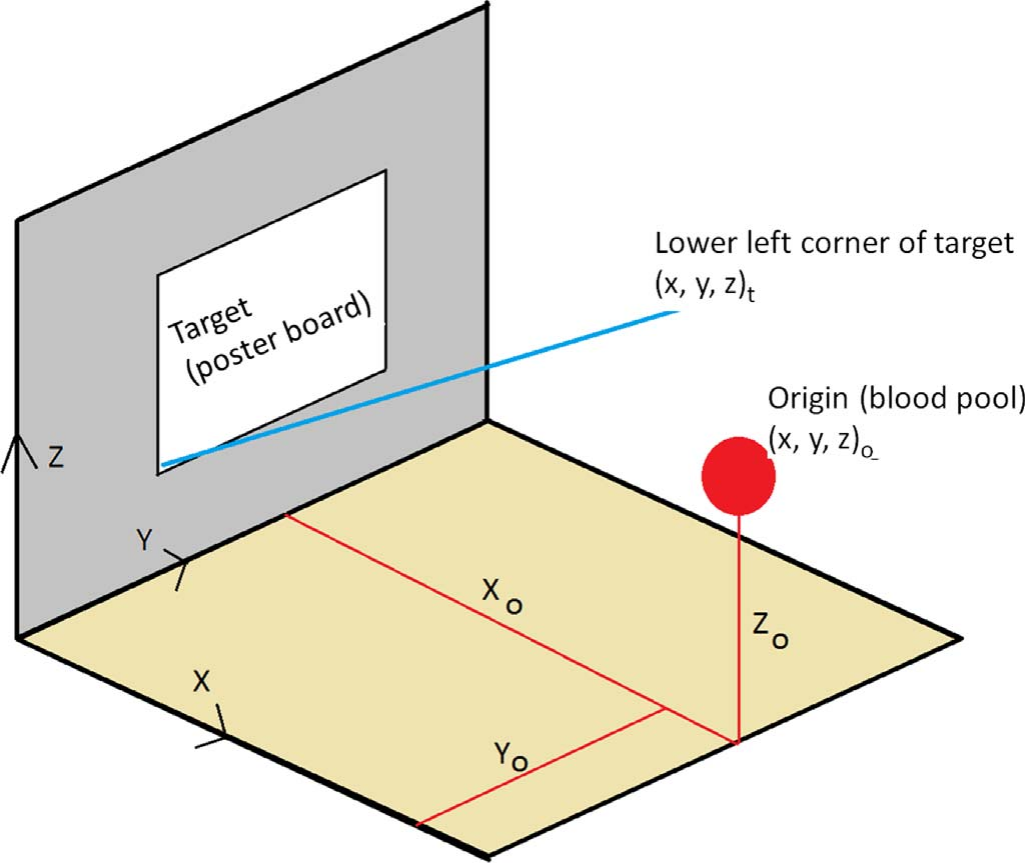
Coordinate system used in the experiments.

The coordinates of the blood origin and the target on which the spatter occurs were measured, as per the geometry in Fig. 2. The coordinate system originated from the bottom left corner of the wall supporting the target. In selected experiments, two blood spatters were generated on the same target, from two different locations, called regions of origin.

**Fig. 3:**
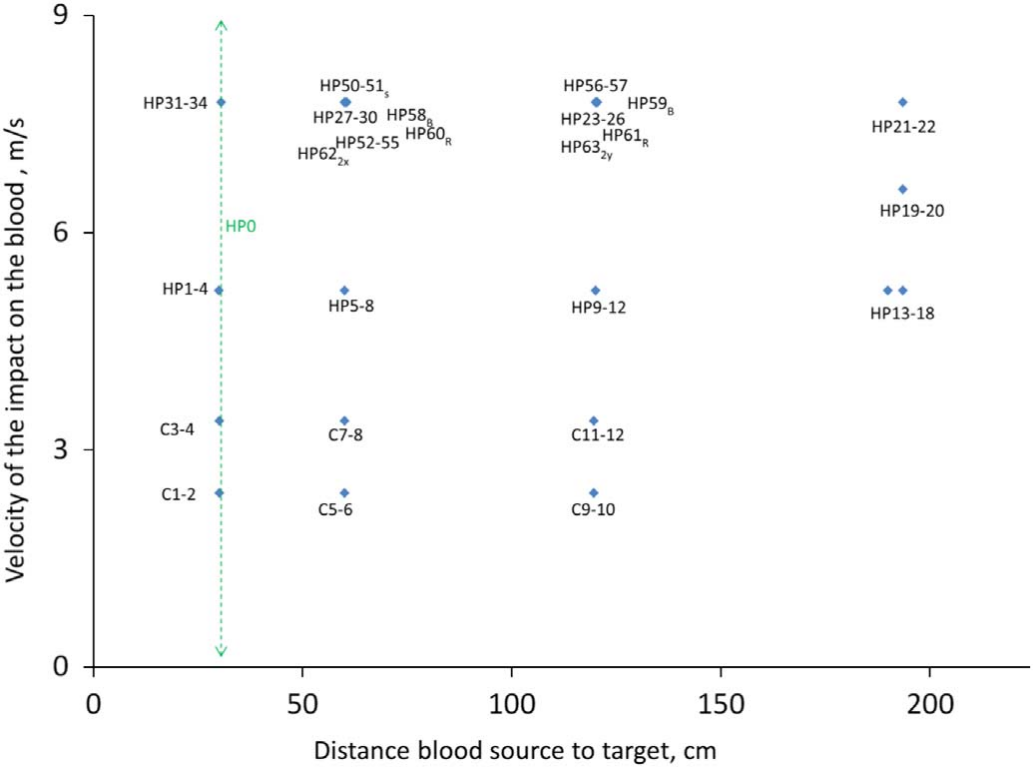
Synthetic view of the data set. *X*-axis is the horizontal distance between source and target; *Y*-axis is velocity of the impactor. Specificities of some blood spatters are shown with subscripts. Abbreviations are HP (dowel rig with hockey puck), B (butcher paper), C (cylinders rig), S (soaked foam at origin), R (rougher side of poster board), 2y (two impacts at different y-locations), 2x (two impacts at different x-locations).

The conditions of each experiment are documented in a text file located in the same directory as the spatter image, and summarized in Table 1, with the range of each parameter, and the reason for documentation of each parameter. Fig. 3 provides a synthetic view of the main variables investigated, the velocity of the impact that atomized the blood, and the distance between blood source and spatter target.

**Table 1:** Description of the variables documented. Ranges of parameters are indicated in brackets, and parameters that have been systematically varied during the investigation are in bold. Standard values of parameters are underlined.

**Parameter and unit**	**Reason for documentation**	**Range or typical values, and uncertainty**
Test rig parameters	Controls the geometry, speed and energy of the blunt impact that generates the spatter	Blood hit between a flat surface and either a cylindrical rod or a parallel flat surface. Impact velocities of 2–8 m/s.
Location of target (x_t_, y_t_, z_t_) and of region of origin (**x**_**o**_, y_o_, z_o_), cm	Spatial information needed for reconstruction	**x**_**o**_–**x**_**t**_**= [30–193.5]**
		z_o_ = 82.5, 155.1
		± 1 cm
Image Scale, DPI (dots per inch)	Spatial information needed for reconstruction	600
Ambient T, °C	Blood physical properties depend on temperature [10]	[20–27] ±1
Relative humidity, %	Evaporation rates [11]	[26.3–63] ± 5
Hematocrit, %	Blood physical properties depend on hematocrit [10]	[36–43] ± 1
Type of target	Controls the spreading, deformation and splashing of the drops [10], [12], [13], [14].	Smooth poster board (1.2/1.56), rougher poster board (3.22/4.19), butcher paper (5.8/7.35). In parenthesis is Ra/RMS roughness, both in μm.
Blood source	Might influence atomization	Pool (1 mL ± 0.2 ml), or foam soaked in blood

## 2 Experimental design, materials and methods

**Fig. 4:**
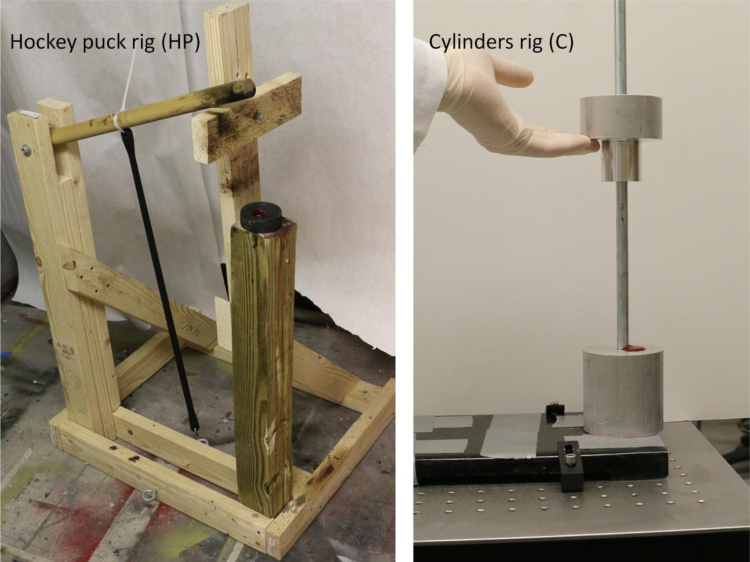
Two test rigs were designed to generate the impact beating blood spatters. Left, a dowel hits a hockey puck where the blood is located. Right is a sliding cylinder device that squeezes the blood between two flat surfaces. In both devices, the initial position of the impactor controls the impact velocity. For scale information, the bottom cylinder on the right is about same diameter as a hockey puck, 7.6 cm.

Two tests rigs were used to create the impact blood spatters, as shown in Fig. 4. In the first rig (dowel and hockey puck, named ‘HP#’), blood is rapidly squeezed between a cylindrical wood dowel (rod) and a flat surface. The dowel rotates by the force of a rubber bungee, and hits a hockey puck where the blood is located. Before impact, a rotating wood slab holds the dowel under tension, at an initial angle controlled by a set of discrete dowel holes around which the slab is free to rotate. That initial position isreported as the dowel height station and numbered 1–3 (3 corresponding to the highest height and tension used in this data set). In the second rig, that with cylinders, named ‘C#’, blood is squeezed between two flat surfaces. The immobile bottom cylinder is 7.7 cm in diameter. The top cylinder is 3.8 cm in diameter, and able to freely fall along a vertical guide rod from a pre-set height.

In the hockey puck rig, the impact contact occurs along a line; in the cylinders rig, across the flat surface of the top cylinder. The measured impact velocities are reported in Table 2. On both rigs, the pool of blood has approximate volume of 1 mL blood, and thickness of 1 mm. To ensure reproducible surface and wettability conditions, the impacting surfaces were rinsed with isopropanol, then deionized water, and dried before each trial. In selected experiments, a soaked polyurethane foam was used instead of a blood pool.

**Table 2:** Measurement of the velocities in experiments, from high-speed movies analysis.

Velocity, m/s	Experiment
2.4 ± 0.1	Cylinder, 30 cm height
3.4 ± 0.1	Cylinder, 60 cm height
5.2 ± 0.2	Rod, dowel position #1
6.6 ± 0.2	Rod, dowel position #2
7.8 ± 0.2	Rod, dowel position #3

The experiments were performed in a closed room (159.2 cm × 249.8 cm × 242.6 cm), where the air was quiescent. Room temperature and relative humidity were measured with a Mannix PTH8708 temperature-humidity pen.

The experiments utilized ethically-sourced swine blood with heparin to prevent coagulation. The blood was drawn less than 48 h prior to any experiment. The blood was placed on a rocker and was at room temperature. Hematocrit was measured with a dedicated centrifugation device (STI, HemataStat-II).

The choice of swine blood can be explained as a compromise between safety and relevance to BPA in a public university laboratory. Indeed, human blood is a biohazard, requiring extensive testing and handling precautions to avoid risks of HIV (human immunodeficiency virus) and hepatitis B & C, which can be deadly if untreated. Artificial blood is still in a development phase, and it is not clear if it will ever be able to match all the complex - and still partly unknown [15]- characteristics of actual blood [3]. Among animal blood, swine blood is the closest to human blood in terms of comparable physical properties [16], such as hematocrit range, shear viscosity of whole blood and plasma, erythrocyte aggregation behavior. Since swine blood has not been associated with risks of HIV or hepatitis B, it is a safer substitute to human blood. Thus, swine blood was drawn from healthy pigs screened for zoonotic diseases at the Ames USDA facilities. Blood was stored refrigerated when not in use and allowed to reach room temperature before use. Personal protection for biohazard including coveralls, gloves, face shield, surgical mask, goggles, were used while producing spatters, and gloves while manipulating dried spatters during e.g. scanning.

Most blood spatters were generated on a target made with flat poster board sheets (UCreate, Walmart Inc., 22 in × 28 in) taped together. The smooth side of the sheet was typically used. Few experiments were made on the rougher side of the same sheets, and other on less expensive butcher paper. Targets of large sizes (up to 136 × 110 cm) were assembled by taping together the back of two or four poster board sheets with masking tape. An optical profilometer Zygo Newview 6300 measured the roughness of the target, with results reported in Table 1. Spreading correlations, which link drop sizes, stain sizes and impact velocities together, have been characterized on those substrates according to the methods in [10]. Note that Table 3 of [10] reporting values of roughness for a range of substrates, has a typo for the roughness, mentioning [mm] instead of the correct magnitude in [μm].

The spatters were scanned at 600DPI (dots per inch) on a flatbed scanner (Epson Expression 11000XL), which is slightly better than what has been obtained with high-end photography [4], and allows to have a clear definition of the edges of most stains. The use of a scanner avoids issues of parallax, which are often present on crime scene photographs. The spatters were scanned in a piecewise manner, by cutting the tape joining the poster board sheets, because the maximum scanning area of the scanner was A3 (297 × 420 mm), significantly smaller than the largest target posters (1360 × 1100 mm). Poster board sheets were never cut in that process. Scans were assembled using image processing software Adobe Photoshop®, and saved as high-quality jpg. A sticker was placed at the center of each sheet, to allow precise assembly of the scans. Photoshop was used to remove marks that were not stains, such as sticker, tape, or pencil marks.
